# A practical topological insulator saturable absorber for mode-locked fiber laser

**DOI:** 10.1038/srep08690

**Published:** 2015-03-03

**Authors:** Peiguang Yan, Rongyong Lin, Shuangchen Ruan, Aijiang Liu, Hao Chen, Yuequn Zheng, Sifan Chen, Chunyu Guo, Juguang Hu

**Affiliations:** 1 Shenzhen key laboratory of laser engineering, College of Optoelectronic Engineering, Shenzhen University, ShenZhen, 518060, China; 2 College of Physics Science and Technology, Shenzhen University, Shenzhen Guangdong 518060, China

## Abstract

A novel saturable absorber (SA) was fabricated by coating the topological insulator (TI) film on microfiber using pulsed laser deposition (PLD) method. The TISA device had an insertion loss of ~1.25 dB, a saturable intensity of 26.7 MW/cm^2^, a modulation depth of ~5.7%, and a nonsaturable loss of 20.5%. Upon employing this SA device, we established a passively mode-locked EDFL and achieved nearly free-chirped soliton pulse with 286 fs of pulse duration and >73 dB of signal to noise ratio (SNR). This result clearly evidences that the PLD is an effective scheme for practical SA device fabrication.

New and high-performance saturable absorbers (SAs) are one of the hottest research topics in ultrafast photonics. Among the various types of SAs, the semiconductor saturable absorber mirrors (SESAMs), carbon nanotubes (CNTs), and graphene were intensively investigated. The SESAMs are regarded as one mature SA with widely application in commercial laser system. But SESAMs are expensive for fabrication and have limited bandwidth operation (typically few-tens nm)^1^. CNTs^2^,^3^,^4^,^5^,^6^ and graphene^6^,^7^,^8^,^9^,^10^,^11^,^12^ have merits of fast recovery time, low saturation intensity and low cost. In mode-locking, CNT is an intrinsic selective-broadband SA depending on its tube-diameter, which usually leads to a larger non-saturable loss for obtaining a broadband operation. Unlike CNT, graphene is a kind of two-dimentional Dirac-material with unique zero bandgap that endows graphene with ultra-broadband absorption property. It has already been used for ultrashort pulse generation in broad wavelength range, spanning from 800 nm to 2500 nm^13^,^14^. But the modulation depth of graphene is often low (typically ~1.3% per layer)^6^. Therefore, significant efforts have been focused on developing new SA beyond graphene. Most recently, a series of Dirac-materials called topological insulators (TIs)^15^,^16^ emerge, such as Bi_2_Te_3_, Bi_2_Se_3_ and Sb_2_Te_3_. These TIs have typical characteristics with a small band gap in the bulk state and a gapless metallic state in the edge/surface. It has been proved that TIs have broadband saturable absorption features and giant third order nonlinear optical property^17^,^18^. Therefore, TISAs attract many attentions as a kind of prominent photonic device for mode-locking.

For a practical SA device, it should have the following fundamental merits: 1) Effective, 2) Robust, 3) Low cost and suitable for mass production. So far, several fabrication method, including of molecular beam epitaxy (MBE) method, mechanical exfoliated method and liquid-phase exfoliation (LPE) method, have been implemented to fabricate TIs^19^,^20^,^21^,^22^,^23^,^24^. Usually, the MBE method can grow high quality film on bulk substrates. For applying in fiber laser system, the film should be translated on fiber ferrule or on side-polished fiber (SPF). Also, the MBE method requires expensive equipment. As a contrast, the mechanical exfoliation method can obtain low-cost thin film by using scotch tape to repeatedly peel the particles from the surface of the bulk crystal. However this method has the lowest repeatability. The LPE method is intensively applied to obtain low-cost few-layer nanomaterials. Note that the nanomaterials are usually doped into thin polyvinyl acetate (PVA) film and hosted on the fiber ferrule in laser cavity. However, this type of SA is vulnerable to optical damage by high power and mechanical damage by fiber connector, and its inherently short nonlinear interaction length could restrict the pulse shaping ability of SA.

The SA device based on the evanescent wave interaction is an effective approach to achieve mode-locked fiber laser^25^,^26^. Recently, a 600 fs erbium-doped fiber laser (EDFL) and a 403 fs thulium-doped fiber laser were achieved by pasting the bulk Bi_2_Te_3_ on SPF^27^,^28^. Subsequently, J. Sotor reported 270 fs and 128 fs EDFL based on the bulk Sb_2_Te_3_ and SPF^29^,^30^. The 270 fs result was the shortest pulse duration without any outer cavity compression so far, but the 128 fs result was obtained after compression with 140 m single mode fiber (SMF). Note that such type of SA device needed the index match oil to induce evanescent wave interaction, and exhibited strong polarization dependent loss. Additionally, because the bulk TI was pasted on the SPF, this SA device was not easily packaged. Another work utilized the evanescent wave to capture Bi_2_Te_3_ nanosheets on microfiber side and produced 1.22 ps pulse^31^. However, such SA device was frangible with high insertion loss (*IL*: ~5.23 dB) and the nanosheets was easily disaffiliated from the microfiber.

As is well-known, pulsed laser deposition (PLD) method is a mature approach to obtain thin film by focusing the high energy laser beam onto the target in vacuum chamber and depositing the vaporized plasma plume on subtract. The film thickness can be controlled by changing the deposition parameters, including of laser pulse energy, temperature and depositing time^32^. Therefore, deposition of the TI film on microfiber would create an effective SA, which has combined advantages from the strong nonlinear optical response in TI material together with the sufficiently-long-range interaction length in fiber taper. Additionally, considering that the PLD film is tightly ‘grown' on microfiber, this type of SA is very robust after packaged. Furthermore, TI film can be deposited on many pieces of microfibers in one time using this method. Hence, this method is low cost and suitable for mass production. However, the fabrication and characteristics of this type of SA, and their application in mode-locking, is yet lacking according to our knowledge.

In this paper, we firstly utilized the PLD method to fabricate a microfiber-based Bi_2_Te_3_-film SA device with the above mentioned merits. The saturable intensity, modulation depth and non-saturable loss were 26.7 MW/cm^2^, ~5.7% and 20.5%, respectively. Upon employing this SA device, we established a passively mode-locked EDFL and achieved nearly free-chirped soliton pulse with 286 fs of pulse duration and >73 dB of signal to noise ratio (SNR). Our results provide a practical photonic device for passively mode-locked fiber laser.

The fabrication process could be divided into two steps: fused-tapering and PLD depositing. In step 1, the single mode fiber (SMF-28 fiber) was tapered to microfiber as shown in Fig. 1(a). The tapered waist had a length of 0.8 mm and a diameter of ~50 μm. In step 2, the Bi_2_Te_3_ target had a diameter of 49 mm and a thickness of 3 mm. The purity of Bi_2_Te_3_ in target was up to 99.999%. The elemental composition was provided by the mat-world.com, in which the bismuth (Bi) had a mass percentage of 52.185% and the tellurium (Te) had a percentage of 47.815%. [Here, Bi has an atomic weight of 208.98 and Te has an atomic weight of 127.6. For the 2:3 ratio of Bi to Te atoms (*e.g.* Te-Bi-Te-Bi-Te), their elemental mass percentages can be deduced]. The laser source was a high energy Nd:YAG laser (SL II-10, Surelite) with 2 W average power and 10 Hz repetition rate. The laser beam was focused on a Bi_2_Te_3_ target, which was placed inside a vacuum chamber with vacuum degree of 5 × 10^−4^ pa. The inspired plasma plume was deposited on microfiber for 90 minutes. The deposition temperature was settled at room temperature.

Figure 1(b) shows the leaky field along the TI-film-coated taper region recorded by a splicer (FSM-60S) by injection of supercontinuum light (Ylsphotonics, SC-5-CFS). The lower part was the face toward plasma plume, while the upper part was the sheltered. It evidenced that the light could really penetrate into the TI film, which in turn gave a modulation of light along the taper region. Fig. 1(c) shows this thin layer of TI film measured by scanning electron microscope (SEM). The thickest location was ~670 nm. Fig. 1(d) was one enlarged region of TI film. It can be seen that the film surface had an appearance like tiny crystal grains, which was quite different from the nano-structured^22^ or bulk TI^27^,^28^,^29^,^30^.

For convenient measuring the elemental stoichiometry and Raman spectrum, we also fabricated the Bi_2_Te_3_ film on quartz glass under the same PLD condition. A energy dispersive spectroscopy (EDS, Oxford 7582) was utilized for the elemental stoichiometry of the as-prepared sample. The EDS analysis in Fig. 2(a) gave that the mass ratio of Bi and Te were 58.27% and 41.73%, respectively. Because that the low-mass Te was evaporated more easily than Bi in the PLD process, the percentage of Te in film was slightly lower than the initial value of 47.815% in target, suggesting that the main elemental component in film was still Bi_2_Te_3_. The Raman spectrum was measured by using a Raman spectrometer (LabRAM HR Evolution) with a laser at 514 nm. A typical laser power of 5.6 mW was used to excite the Raman scatting. Fig. 2(b) shows the measured Raman spectrum. Three Raman optical phonon peaks was observed at E_g_^2^ of 101 cm^−1^, A_1u_^2^ of 122 cm^−1^ and A_1g_^2^ of 140 cm^−1^. All these peaks were known to exist in the Bi_2_Te_3_ crystal^33^.

To form a compact device, the film-coated microfiber was packaged into a heat-shrink tube as shown by the inset in Fig. 3(a). The insertion loss (IL) of this device was measured to be ~1.25 dB. Its linear absorption was measured from 1500 nm to 1600 nm by using a supercontinuum source (Ylsphotonics, SC-5-CFS) and optical spectrum analyzer (OSA). The linear transmission was characterized by a very flat profile at the level of 75% ± 2%. For comparison, the microfiber without TI film was also provided in Fig. 3(a), which has a flat profile at the level of 83% ± 2.5%. The nonlinear saturable absorption measurement was carried out using a home-made femtosecond laser source (central wavelength: 1560.8 nm, repetition rate: 18.55 MHz, pulse duration: ~140 fs). The measuring method was similar to that in previous works^31^,^34^. The corresponding result was shown in Fig. 3(b), which gave a saturable intensity *I*_sat_ of 26.7 MW/cm^2^, a modulation depth Δ*α* of 5.7%, and a nonsaturable loss *α*_ns_ of 20.5%. For comparison, we listed the typical results of TISA in reported passively mode-locked EDFLs in table 1. The *I*_sat_ and Δ*α* of our TISA were comparable to the 31 MW/cm^2^ and 6% in Refs. 29, 30. It should be pointed out that the low value of *I*_sat_ and Δ*α* was not a defect of SA, which could lower the mode-locking threshold in laser system. A recent case was that the 1.7% of Δ*α* was sufficient for mode-locking^31^. Notably, the *α*_ns_ and *IL* of our SA were great lower than corresponding values of microfiber-based TI nanosheets SA in Ref. 31, indicating that our SA device was more efficient for mode-locking in laser system.

Figure 4 shows the schematic of mode-locked fiber laser with our TISA device. The pump source was a laser diode (LD) with emission centered at 976 nm. A piece of 2.4 m-long EDF was used as the laser gain medium with absorption coefficient of 25 dB/m@980 nm (IsoGain™ I-25, Fibercore). The pump was delivered into EDF via a 980/1550 fused wavelength division multiplexer (WDM) coupler. A polarization dependent isolator (ISO), placed after the EDF, was used to ensure unidirectional operation and eliminate undesired feedback from the output end facet. A fused fiber optical coupler (OC) was used to extract 30% energy from the cavity. A polarization controller (PC), consisting of three spools of SMF-28 fiber, was placed in the ring cavity after the ISO. The TISA was inserted between the PC and the WDM coupler. Apart from the gain fiber, all the fiber devices in cavity were made by SMF-28 fiber. The total cavity length was ~11 m. The cavity length was optimized in experiment. The performance of the laser was observed using an OSA (Yokogawa, AQ6370B), 1 GHz digital oscilloscope (Tektronix DPO7104C), 3 GHz RF spectrum analyzer (Tektronix, RSA3303B) coupled with a 1 GHz photodetector, and an optical autocorrelator (APE PulseCheck).

The mode-locking state appeared at the pump power of 36 mW, and operated stably below the pump power of 54 mW. Fig. 5(a) shows the measured optical spectrum at the pump power of 48 mW. It can be seen that the output spectrum was centered at 1560.8 nm with a 3 dB bandwidth of 9.15 nm. Three pairs of Kelly sidebands were clearly observed on the optical spectrum, indicating that the mode-locked operation was in soliton regime. Fig. 5(b) shows the measured autocorrelation trace of the output pulse together with secant hyperbolic-fitting curve. The pulse duration was 286 fs and the time-bandwidth product (TBP) was 0.322, which was close to the 0.315 of transform-limited sech^2^ pulses. The RF spectrum of the laser was depicted in Fig. 5(c). The fundamental cavity frequency was 18.55 MHz (Corresponding to the cavity round-trip time ~53.9 ns). The electrical signal to noise ratio (SNR) was >73 dB measured with 20 kHz resolution bandwidth (RBW), demonstrating the mode-locking state was quite stable. The inset was a RF spectrum for the 900 MHz scanning range with same RBW. The average output power was measured to be ~0.5 mW at pump power of 48 mW, corresponding to the pulse energy and peak power of 27 pJ and 61 W, respectively.

To check the stability, we changed the pump power and found that the mode-locking state could start each time when the pump was retuned into the power range. Furthermore, a 9-day continuously measurement on the mode-locking state was also carried out at the pump power of 48 ± 0.2 mW, and the results revealed that the central peak locations and 3 dB bandwidths remained relatively stable, as illustrated in Fig. 6. It should be pointed out that the rotation of PC still greatly impacted the mode-locking state as reported in Refs. 31, 34. To verify whether the TISA contributed to the passive mode-locking, we replaced it in the ring laser by the same type microfiber without TI film. In this case, even we carefully adjusted PC orientation and changed pump power in the range from 36 mW to 54 mW, the mode-locking state could not be observed again. This result demonstrated that the mode-locking behavior was caused not by the nonlinear polarization rotation (NPR) effect under the same condition, which testified that the TISA was indeed contributing to the mode-locking operation.

As a summary, a new microfiber-based TISA device was demonstrated by the PLD method with some unique advantages, such as effective, robust, low cost and suitable for mass production. It had an *IL* of ~1.25 dB, a saturable intensity of 26.7 MW/cm^2^, a modulation depth of ~5.7%, and a nonsaturable loss of 20.5%. Based on this SA device, an all-fiber passively mode-locked EDFL was constructed and achieved stable soliton output with 286 fs of pulse duration and >73 dB of SNR. The performance of our SA can be further enhanced by optimizing the taper waist of microfiber and TI film deposition parameters. This experimental result clearly evidences that the PLD is an effective scheme for practical SA device fabrication.

## Author Contributions

**Figure 1:**
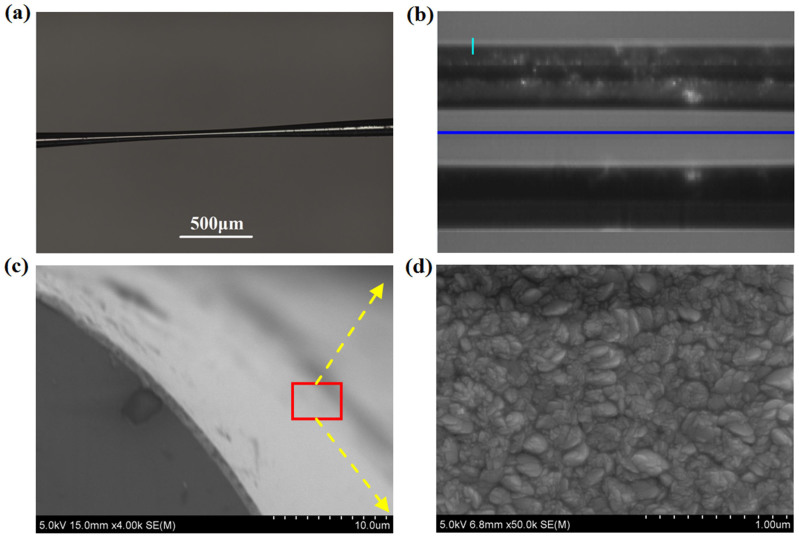
Characteristics of the microfiber coated with TI film, (a) Side view photo; (b) Evanescent field from the damaged region; (c) Film thickness; (d) Enlarged film surface.

**Figure 2:**
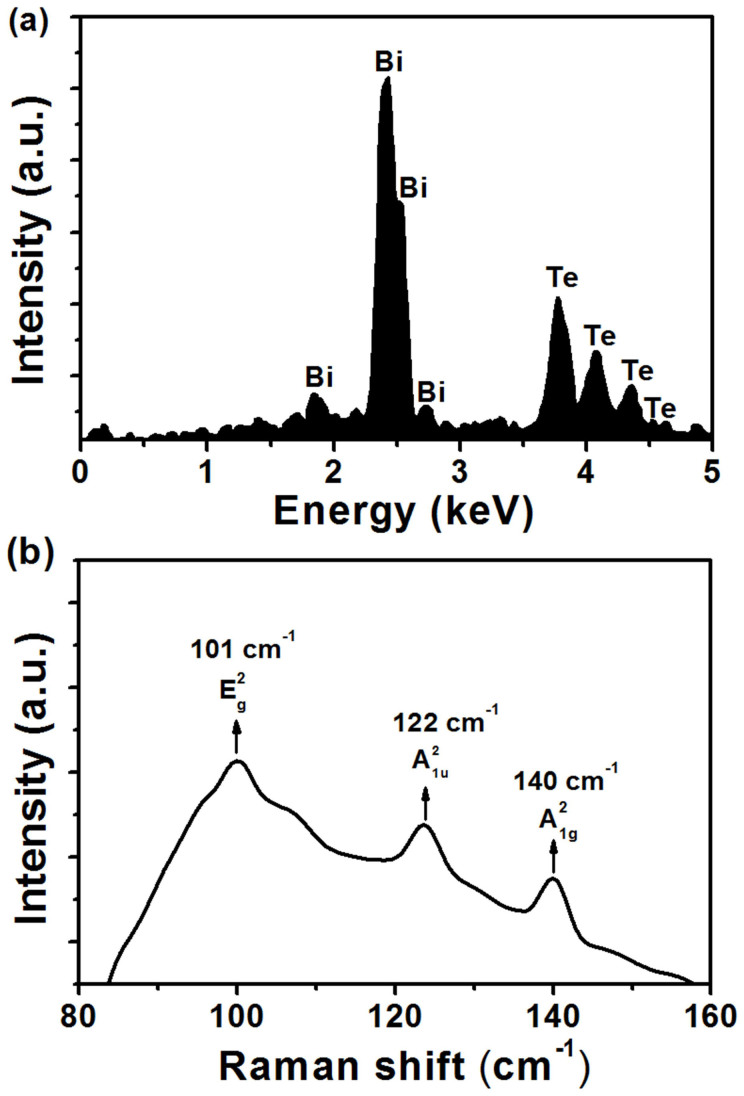
(a) EDS of TI film. (b) Raman spectrum of TI film.

P.G.Y. wrote the main manuscript text and did the whole mode-locking experiment. R.Y.L. fabricated the TISA in experiment. S.C.R. provide experimental equipment and Lab. A.J.L. obtained the SEM in Fig.1. H.C. and Y.Q.Z. prepared the data of Fig.2. S.F.C. measured the Raman spectrum of TI-film. C.Y.G. supplied the microfiber. J.G.H. supplied the P.L.D. equipment. All authors reviewed the manuscript.

## Figures and Tables

**Figure 3:**
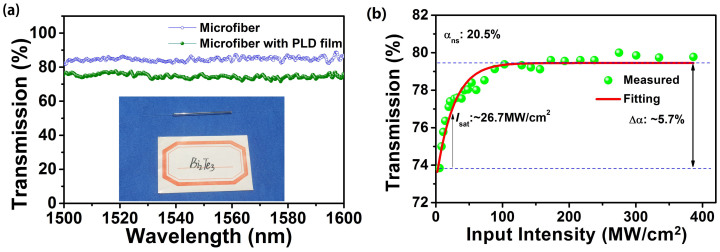
(a) Measured linear absorption (Inset was TISA packaged by heatshrink tube). (b) Measured nonlinear saturable absorption of the microfiber-based TISA device.

**Figure 4:**
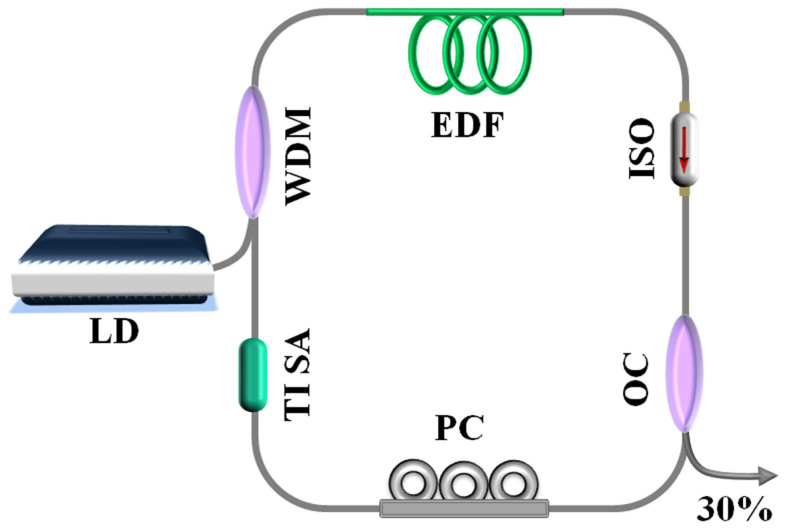
Schematic of mode-locked fiber laser with TISA.

**Figure 5:**
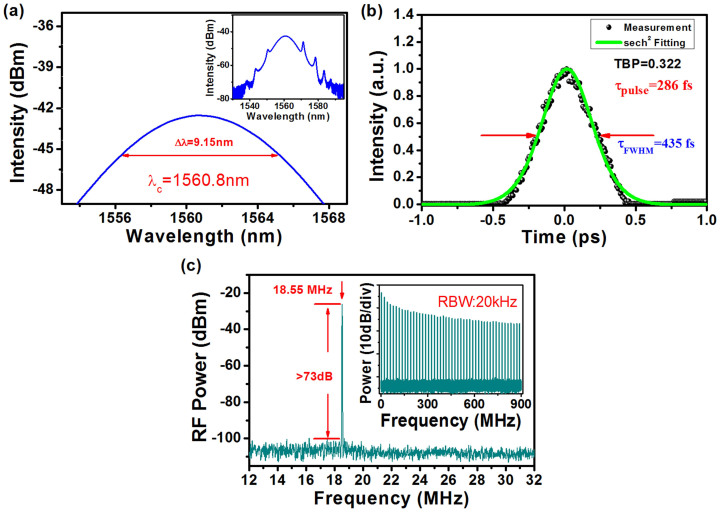
(a) Optical spectrum of the generated soliton pulses, (b) Autocorrelation trace, (c) RF spectrum of the mode-locked laser.

**Figure 6:**
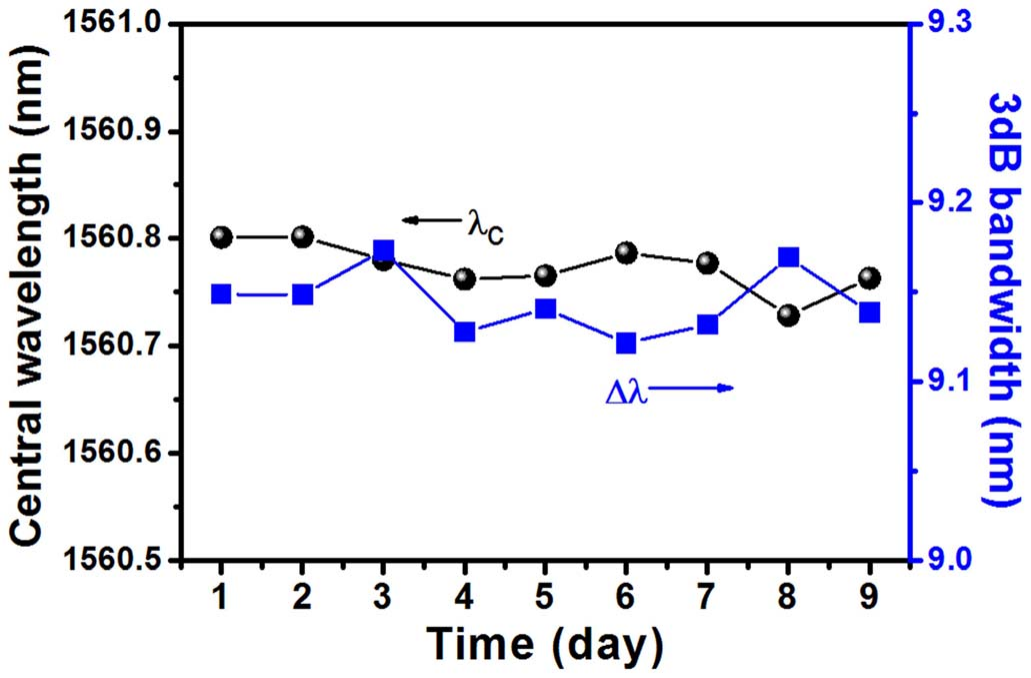
Central wavelength and 3 dB bandwidth of the mode-locked laser measured in 9 days.

**Table 1:** Typical TISA parameters in recent reports

TI SA in ref.	*I*_sat_ (MW/cm^2^)	Δ*a* (%)	*a*_ns _(%)	IL (dB)
Bi_2_Se_3_/PVA film [23]	11	4.3	36.3	~3.62
Bi_2_Se_3_/PVA film [24]	12	3.9	67.5	*
Bi2Te3/SPF [27]	*	15.7	17	1.7
Sb_2_Te_3_/SPF [29]	31	6	43	~3
Bi_2_Te_3_ nanosheets/microfiber [31]	*	1.7	69.9	5.23
Bi_2_Te_3_ PLD film/microfiber **	26.7	5.7	20.5	1.25

*Not available, **This work.
